# Activation of hip prostheses in high energy radiotherapy and resultant dose to nearby tissue

**DOI:** 10.1002/acm2.12058

**Published:** 2017-02-27

**Authors:** Stephanie Keehan, Ryan L. Smith, Jeremy Millar, Max Esser, Michael L. Taylor, Peta Lonski, Tomas Kron, Rick D. Franich

**Affiliations:** ^1^ School of Science RMIT University Melbourne Victoria Australia; ^2^ William Buckland Radiotherapy Centre The Alfred Hosptial Melbourne Victoria Australia; ^3^ Department of Orthopaedic Surgery The Alfred Hosptial Melbourne Victoria Australia; ^4^ Physical Sciences Peter MacCallum Cancer Centre Melbourne Victoria Australia

**Keywords:** high energy radiotherapy, photonuclear effect, prostheses activation

## Abstract

High energy radiotherapy can produce contaminant neutrons through the photonuclear effect. Patients receiving external beam radiation therapy to the pelvis may have high‐density hip prostheses. Metallic materials such as those in hip prostheses, often have high cross‐sections for neutron interaction. In this study, Thackray (UK) prosthetic hips have been irradiated by 18 MV radiotherapy beams to evaluate the additional dose to patients from the activation products. Hips were irradiated in‐ and out‐of field at various distances from the beam isocenter to assess activation caused in‐field by photo‐activation, and neutron activation which occurs both in and out‐of‐field. NaI(Tl) scintillator detectors were used to measure the subsequent gamma‐ray emissions and their half‐lives. High sensitivity Mg, Cu, P doped LiF thermoluminescence dosimeter chips (TLD‐100H) were used to measure the subsequent dose at the surface of a prosthesis over the 12 h following an in‐field irradiation of 10,000 MU to a hip prosthesis located at the beam isocenter in a water phantom. ^53^Fe, ^56^Mn, and ^52^V were identified within the hip following irradiation by radiotherapy beams. The dose measured at the surface of a prosthesis following irradiation in a water phantom was 0.20 mGy over 12 h. The dose at the surface of prostheses irradiated to 200 MU was below the limit of detection (0.05 mGy) of the TLD100H. Prosthetic hips are activated by incident photons and neutrons in high energy radiotherapy, however, the dose resulting from activation is very small.

## 1 Introduction

The photonuclear effect is the interaction of a high energy photon with a nucleus resulting in emission of one or more nucleons. In radiotherapy, medical linear accelerators operating with accelerating potential above ~7 MV produce photons of sufficient energy to interact via the photonuclear effect^1^ and produce secondary neutrons. The presence of secondary neutron contamination in high energy radiotherapy beams is well known.^2^, ^3^ Neutrons may pose a significant risk due to their high radiobiological effectiveness (RBE) of up to 20 for 1 MeV neutrons.^4^

Furthermore, patient implants, pacemakers, or prostheses composed of non‐biological materials may invite significantly different interactions with incident photon and neutron radiation. Modern radiotherapy typically involves highly conformal photon beams directed toward a target volume. Contaminant neutrons may be scattered by collimators, but are not efficiently absorbed by them. Compared to the photon field, neutrons do not exist as a collimated beam, but as a diffuse fluence incident on the entire patient.^5^

It is common practice to avoid directly irradiating metallic prostheses in treatment planning as they are known to perturb the radiation field and reduce the dose to the tissue downstream of the prosthesis.^6^ Treatment planning systems do not accurately account for beams traversing metallic implants and estimate the reduction in dose poorly.^7^ However, beams are commonly allowed to pass nearby or even through prostheses on exit from the patient.

Prostheses may be activated by photons and/or neutrons in high energy radiotherapy. High energy photons can activate prostheses *via* the photonuclear effect; causing the emission of neutrons from within the patient. Neutrons produced in the linac can also activate the prostheses, even when planned treatment beams are not directly incident upon them, due to the diffuse nature of the neutron contamination arising from the high frequency of neutron scattering events. These neutrons have mean energies in the vicinity of 0.14 MeV in tissue^8^ and, as such, have high cross‐sections for capture with the nuclei of hip prostheses. Neutron capture often induces radioactivity in the target nuclei.

In addition to the direct interaction of neutrons with patient tissue, neutrons pose another potential exposure pathway to patients *via* the production of unstable product nuclei following neutron capture. These radioactive nuclei may emit secondary radiations with half‐lives that may be much longer than the beam irradiation times. We have previously reported on the isotopes, half‐lives, and doses resulting from activity produced in components of linacs operated at high energies.^9^ In the present work, we extend this to considering the neutron activation of metal objects *inside* the patient. The decay of activation products may involve radiation emissions that will deposit energy in nearby tissue.

We have investigated the activation of metallic hip prostheses by 18 MV radiotherapy photon beams which are commonly used for these treatments. Prostheses were irradiated in water phantoms in a number of geometries, both in‐ and out‐of direct photon beams. Gamma‐ray spectroscopy was used to identify the isotopes and half‐lives produced within metallic hip prostheses. The resultant doses at the surface of a prosthesis were measured with high sensitivity TLD100H dosimeters, to address the question of whether these secondary doses might be significant.

## 2 Method

Prosthetic hips (Thackray, UK) were irradiated in and out‐of‐field by 18 MV photon beams in a water phantom in different geometries. The isotopes induced and their half‐lives were identified through gamma ray spectroscopy. The dose at the surface of an irradiated prosthesis was also measured using high sensitivity thermoluminescence dosimetry (TLD‐100H).

### 2.A Irradiation

**Figure 1:**
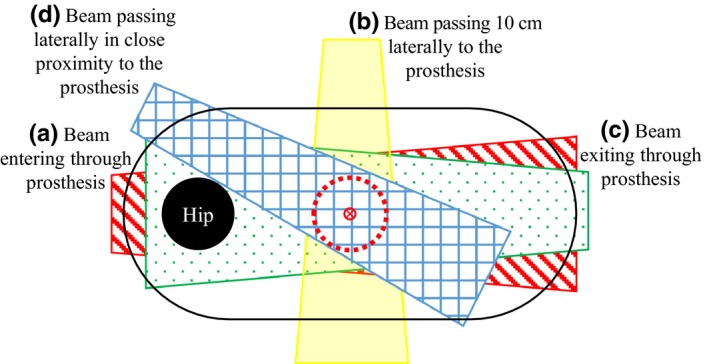
Simulated patient irradiation geometries. Beam (a) enters through the prosthesis, (b) passes laterally 10 cm away. Beam (c) exits through the prosthesis with a larger degree of photon attenuation and neutron moderation than beam (a). Beam (d) also passes laterally in close proximity (within 1 cm) to the prosthesis. The labels (a), (b), (c), and (d) correspond to the geometries shown in Fig. 2.

**Figure 2:**
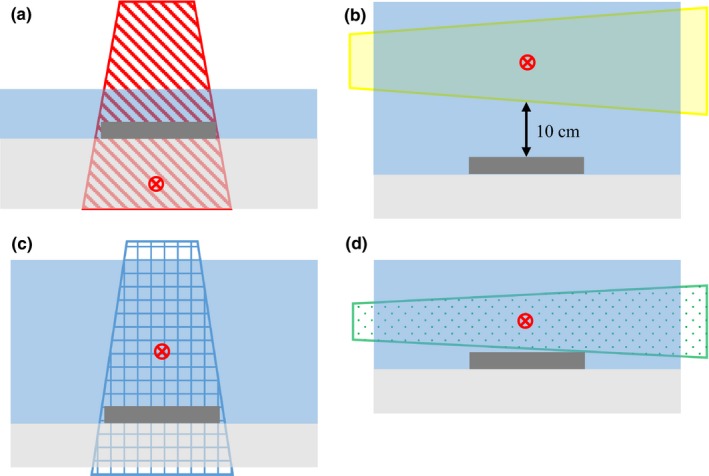
Prosthetic hips were irradiated in a water tank by (a) a beam entering through the prosthesis, (b) a beam passing 10 cm laterally to the prosthesis, (c) a beam exiting through the prosthesis, and (d) a beam passing laterally in close proximity to the prosthesis. The red circle represents the isocenter of the beam. Each water phantom was placed on at least 3 cm of solid water backscatter material. The labels (a), (b), (c), and (d) correspond to the geometries shown in Fig. 1.

The four representative irradiation geometries of interest are shown in Fig. 1. The beam entering through the prosthesis (Fig. 1a) should ideally be avoided clinically because the prosthesis directly shadows the target. However, this geometry is sometimes necessary to avoid adjacent organs at risk. It is also of interest as it provides a “worst‐case” scenario for activation. The other three geometries are more commonly encountered clinically. The beam exiting through the prosthesis, Fig. 1(c), is identical to the “entry” beam (a) except the beam has been significantly attenuated before entering the prosthesis. Beams (b) and (d) pass laterally to the prosthesis, which is exposed only to out‐of‐field photons and neutrons scattered from the linac head. The water phantom configurations used to simulate these geometries are shown in Fig. 2(a–d). Each water phantom was placed on at least 3 cm of solid water backscatter material. Each irradiation was 200 MU delivered by a Varian 21 EX at a dose rate of 400 MU/min in 18 MV (TPR_20,10 _= 0.784) photon mode.

### 2.B Gamma‐ray spectroscopy

Acquisition of the gamma ray energy spectra was initiated within 1 minute of termination of the beam with a 3 × 3″ NaI(Tl) scintillation detector (Saint‐Gobain, France) and an Ortec DigiBASE‐E Ethernet multichannel analyser PMT base (Ametek, USA). The spectra were each acquired for 15 min to allow sufficient counts for peak identification.

### 2.C Thermoluminescence dosimetry

**Figure 3:**
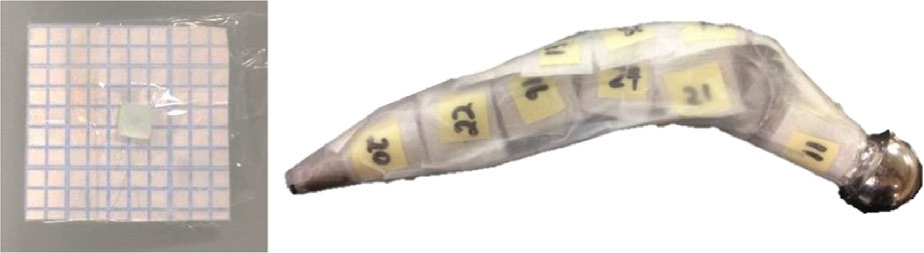
The left figure shows a 3 × 3 mm TLD‐100H chip. It is affixed to a small square of graph paper by plastic cling wrap which is held in place by double sided tape at the edges of the square. The right figure shows the graph paper squares with attached TLDs affixed to the hip prosthesis following irradiation.

To induce sufficient activity to produce a measurable dose, one prosthesis was irradiated at isocenter in water by 10,000 MU from an Elekta Synergy at a dose rate of 450 MU/min at 18 MV (TPR_20,10_ = 0.780). High sensitivity LiF:Mg,Cu,P thermoluminescence dosimeters (TLD‐100H) (Harshaw, USA) were used to measure the dose resulting from radioactive isotopes produced in the prosthesis. 24 TLD chips (0.89 × 3.1 × 3.1 mm^3^) were placed on the surface of the hip within 2 minutes of termination of the beam (see Fig. 3) and removed after 12 h (5 half‐lives of the longest lived isotope). The responses of the TLDs were measured with a Harshaw 5500 automated reader. Each TLD was corrected for its individual sensitivity relative to the batch average as determined in a 6 MV beam.^10^

The average signal of 16 un‐irradiated TLDs was subtracted from the signal of TLDs placed on the hip to remove background signal unrelated to the irradiation. This is important for reducing uncertainty when using high sensitivity TLDs to measure very low doses.^11^ The backgound dose was minimized by annealing the TLDs immediately before the measurements were performed and the read‐out was within 24 h of the measurements. A total of 50 TLD chips were read out with a Harshaw 5500 automatic reader; a process which takes approximately 1 h. Half of the un‐irradiated background TLDs were read at the beginning and the other half at the end to allow for variations over the duration of the read‐out session. Standards irradiated to 5, 10, 20, and 50 cGy at 6 MV were also read during the session for calibration of the dosimeters affixed to the prosthesis. Almost 6 MV was used to calibrate the TLDs to avoid an over response due to the small concentration of^6^ Li present in TLD100H and the contaminant neutrons present in 18 MV beams.^12^

### 2.D Film dosimetry

One prosthesis was irradiated in a phantom with 10 cm of water build up. The hip was placed at the center of a 40 × 40 cm^2^ field and irradiated by 10, 000 MU from a Varian 21EX at 18 MV (TPR_20,10_ = 0.784). The prosthesis was placed on a piece of Gafchromic XR QA film, with sensitivity 0.2–50 cGy, within 2 minutes of completion of the irradiation and remained there for 72 days.

## 3 Results

**Figure 4:**
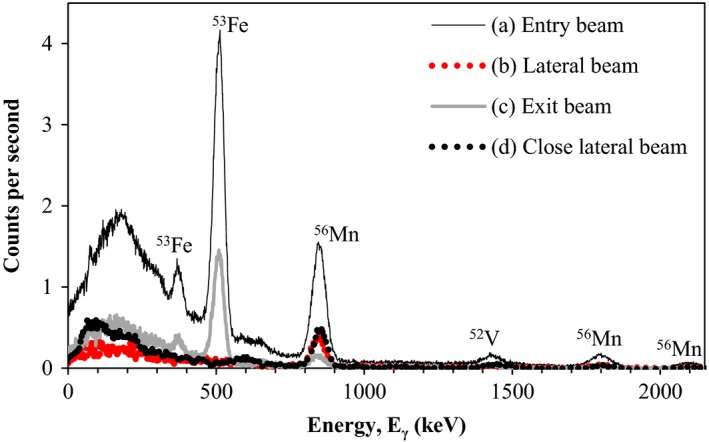
Gamma‐ray energy spectra acquired over 15 min within one minute of irradiation, for each of the irradiation schemes illustrated in Figs. 1 and 2.

The gamma‐ray spectra acquired following irradiation show the characteristic gamma‐ray peaks of ^53^Fe, ^56^Mn, and ^52^V (Fig. 4). The shape of the spectrum at the low energy end is due to Compton scattering and over lapping back scatter peaks. The total count rates measured during spectroscopy of in‐field irradiated prostheses were between 6 and 7.5 times higher than those measured from out‐of‐field irradiated prostheses. The prostheses irradiated in the lateral out‐of‐field region are notable for the absence of the ^53^Fe gamma‐ray peaks. The half‐lives and gamma‐ray energies of these isotopes are given in Table 1.

**Table 1:** The gamma‐ray energies and half‐lives of the isotopes produced within the hip prostheses

E*γ* (keV)	t_1/2_ (min)	Isotope	Reaction
378, 511	8.51	^53^Fe	(*γ*,n)
847, 1811, 2113	155	^56^Mn	(n,*γ*)
1434	3.74	^52^V	(n,*γ*)

There may be additional positron emitting isotopes contributing to the photopeak observed at 511 keV. We are able to identify ^53^Fe as a contributor because of its additional gamma emission of 378 keV. Other isotopes without additional gamma emissions of significant intensity, produced by incident photons *via* the photonuclear effect, may be present, but cannot be identified without further evidence of their presence.

**Figure 5:**
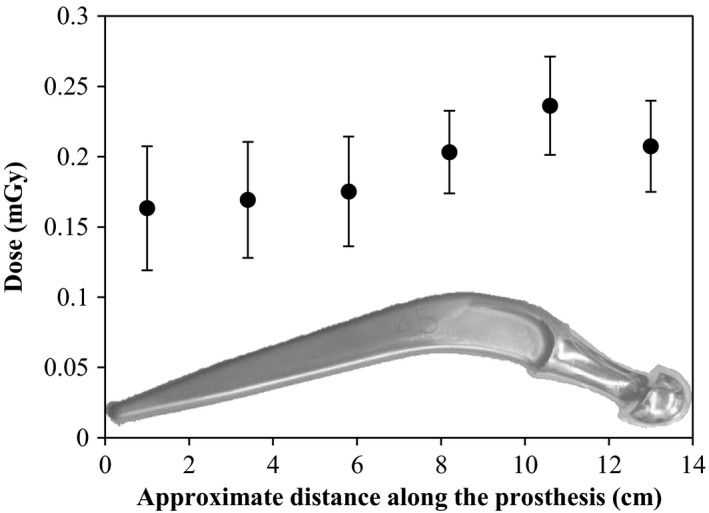
The dose measured on the surface of the prosthesis as a function of the approximate distance from the narrow end to the thicker end which is inserted into the socket. The error bars are the standard deviations of three to four TLD measurements taken at approximately the same distance. Inset: Photo of the hip.

The average dose measured over the 12 h immediately following direct irradiation of 10,000 MU was 0.20 mGy at an effective distance of 1 mm (LiF density 2.2 g/cm^3^, thickness of the chip 0.89 mm) from the surface with a standard deviation of 0.04 mGy. The limit of detection calculated from the mean and standard deviation of the readings of the un‐irradiated background TLDs was 0.05 mGy (with a 99% confidence interval). The highest dose was recorded by the TLDs attached to the thickest part of the prosthesis reflecting the proximity of greater mass of activated material (see Fig. 5). The gafchromic film showed no measurable response after 72 days in contact with an irradiated prosthesis indicating the dose rate for long lived isotopes would be well below 2 μGy/h after an exposure of the prosthesis to 100 Gy of 18 MV X‐rays”.

## 4 Discussion

Radioactive ^53^Fe with a half‐life of 8.51 min was observed only in prostheses directly irradiated by 18 MV photon beams. Production of ^53^Fe is thus attributed to photonuclear interactions with stable ^54^Fe and involves neutron production from within the prosthesis itself. The absence of ^53^Fe from prostheses *not* directly irradiated implies it is produced by photons (in photonuclear interactions) and not by neutrons. This neutron production from within the patient may pose a further risk because of the high relative biological effectiveness (RBE) of neutron radiation.

**Figure 6:**
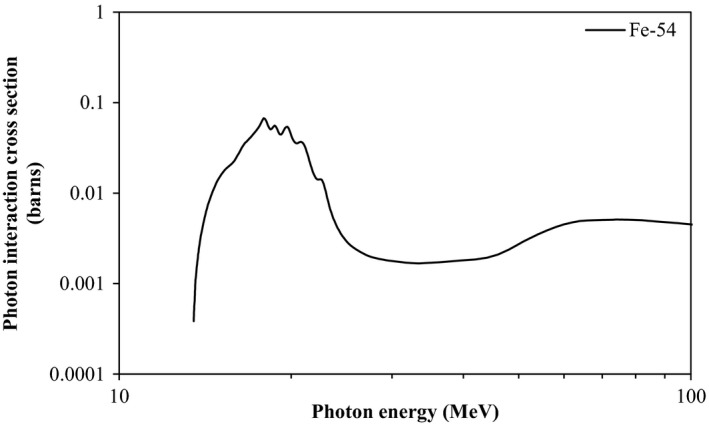
The cross‐section for neutron production by photons with ^54^Fe nuclei as a function of photon energy. These data are from the JENDL 4.0 nuclear data library.^16^

The scattered photons in the out‐of‐field region generally have lower energy than those in the primary field. The threshold for the ^54^Fe(*γ*,n) interaction is 13.5 MeV (see Fig. 6). It is possible that a small number of scattered photons with sufficient energy reach prostheses out of field, but there are far fewer photons and they generally have lower energies than those in field. These two factors combined result in a much lower overall probability of the interaction occurring and the production of ^53^Fe out of field was not observed.

^56^Mn and ^52^V with half‐lives of 155 and 3.74 min, respectively, were observed for prostheses irradiated in‐field as well as those placed just outside the field and 10 cm from the field. The production of these isotopes is attributed to activation by contaminant neutrons which do not exhibit the same penumbral drop in fluence as photons.

^56^Mn and ^52^V also produce beta particles with end point energies of 736, 1038, 2540, and 2849 keV.^13^, ^14^ These have maximum ranges in tissue of 0.26, 0.41, 1.18, and 1.44 cm respectively in ICRP tissue.^15^ Any dose which may be contributed by particles escaping the surface of the prosthesis is included in the dose measured by TLDs.

Isotopes with longer half‐lives may have been produced and not identified during gamma spectroscopy because of the relatively high decay rate of the short‐lived isotopes immediately following exposure. Longer lived isotopes can in principle deliver a higher dose, but there was no measurable response in film placed in contact with an irradiated prosthesis for 72 days.

The photon dose measured from two minutes post irradiation for a 12‐hour duration was 0.20 ± 0.04 mGy for a 10,000 MU irradiation. Twelve hours is approximately five times the longest half‐life identified and more than 96% of the radionuclei produced will decay during this time. The dose is slightly higher at the surface of the hip in the thicker regions which is due to the larger mass present. This dose is minute compared to the treatment dose and even the out‐of‐field doses resulting from scatter and leakage x‐rays, which may be of the order of 0.1% or 70 mGy for a typical prostate treatment of 70 Gy.

**Figure 7:**
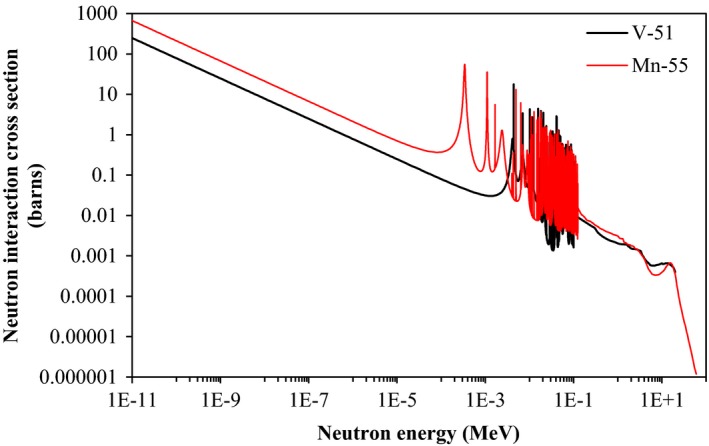
The cross‐sections for radiative neutron capture by ^51^V and ^55^Mn nuclei as a function of neutron energy. These data are from the B‐VII.1 nuclear data library.^1^

The dose rate induced in prostheses following a typical 2 Gy treatment fraction is of greater clinical relevance. The surface dose over 12 h was below the minimum detectable limit of 0.05 mGy following the 200 MU exposures performed to measure the gamma spectra of the activated prostheses. The activity induced in a material is not linearly related to the number of monitor units delivered because some nuclei decay during the beam delivery. The overall activity induced is dependent on the number of MU, the dose rate, and the rate at which the isotopes are produced, which depends on the cross‐sections of the interactions which produce them. These cross‐sections are strongly energy dependent (Figs. 6 and 7) and detailed calculations relating activity from one exposure to another cannot be made without accurate energy spectrum data. The activity of those prostheses irradiated in‐field was much higher than those irradiated out of the primary photon field. The surface dose rate of a prosthesis irradiated out‐of‐field would be expected to be reduced by a similar factor, and it would be below the limit of detection of 0.05 mGy.

## 5 Conclusions

Contaminant neutrons in high energy radiotherapy can induce radioactivity in metallic prostheses, even when prostheses are outside the primary field. ^53^Fe was found only in prostheses directly irradiated by 18 MV radiotherapy beams. ^56^Mn and ^52^V were present in prostheses directly irradiated and those exposed between ~1 and 10 cm from the edge of the field. ^53^Fe was observed only from in‐field irradiation and is therefore attributed to the photonuclear effect. Since ^56^Mn and ^52^V are produced regardless of whether the prosthetic hip was in‐ or out‐of the photon field, they are attributable to neutron activation. The dose measured at the surface of a prosthesis irradiated to 10,000 MU was 0.20 mGy over 12 h (five half‐lives of the longest lived isotope produced in the prosthesis). The surface dose rates for fewer MU and out‐of‐field irradiations were below the limit of detection for the TLDs. This is very low dose when compared to prescribed radiotherapy doses and even the out‐of‐field photon dose a patient receives from scatter and leakage radiation.

## Conflict of Interest

None.
